# Pericardial malignant mesothelioma diagnosed in a dog by immunocytochemistry of the pericardial fluid: a case report

**DOI:** 10.1186/s12917-023-03655-8

**Published:** 2023-07-21

**Authors:** Eun Wha Choi

**Affiliations:** Department of Veterinary Clinical Pathology, College of Veterinary Medicine & Institute of Veterinary Science, Kangwon National University, 1 Kangwondaehak-gil, Chuncheon, 24341 Gangwon-do Republic of Korea

**Keywords:** Pericardial effusion, Mesothelioma, Immunocytochemistry, E-cadherin, Calretinin

## Abstract

**Background:**

Pericardial effusions are one of the most common cardiac diseases in dogs. Common causes of haemorrhagic pericardial effusions include neoplasia, such as hemangiosarcoma, mesothelioma, chemodectoma, and ectopic thyroid tumours, and benign idiopathic pericardial effusion. Distinguishing among reactive mesothelial cells, malignant mesothelioma, and adenocarcinoma in body effusions is a diagnostic challenge. Therefore, the author aimed to discover whether the observed cells were reactive mesothelial, mesothelioma, or adenocarcinoma cells through immunocytochemistry using five markers (cytokeratin, vimentin, desmin, E-cadherin, and calretinin) in a canine patient.

**Case presentation:**

A 2.1 kg, spayed female, 10-year-old Yorkshire Terrier dog presented to a local hospital with dyspnoea and was evaluated for pericardial effusion. The presence of pericardial fluid was confirmed, and she was referred to our hospital for further evaluation. In cytological evaluation, cells shed individually or in clusters were observed, along with numerous non-degenerative neutrophils and macrophages. The cells showed binucleation, anisocytosis, anisokaryosis, abnormal nucleoli, abundant basophilic cytoplasm, high nuclear–cytoplasmic ratio, and coarse chromatin. Large atypical multinucleate cells were also observed. Erythrophagia was observed, indicating chronic haemorrhage. Immunocytochemistry using pericardial fluid was positive for cytokeratin, vimentin, desmin, E-cadherin, and calretinin. Therefore, malignant mesothelioma was diagnosed.

**Conclusions:**

Immunocytochemistry is a very useful diagnostic technique because it can determine whether several fluorescent markers are simultaneously expressed in the same cell. Further, E-cadherin and calretinin can be used for the differential diagnosis of reactive mesothelial cells, malignant mesothelioma, and adenocarcinoma in dogs.

## Background

Pericardial effusions are one of the most common cardiac diseases in dogs [1]. Approximately 41% of canine pericardial effusions are due to neoplastic causes, 45% due to benign idiopathic pericarditis, and the remaining 14% due to non-neoplastic pericardial diseases [2].

Distinguishing among reactive mesothelial cells, malignant mesothelioma, and adenocarcinoma in body effusions has been a diagnostic challenge [3]. Reactive mesothelial cells show some morphological characteristics of malignancy and are often confused with the neoplastic cells of malignant mesothelioma [4]. Further differentiation between adenocarcinomas and mesotheliomas is another diagnostic challenge in cytology.

Previously, E-cadherin was used to distinguish between reactive mesothelial cells and carcinoma in body effusions, but it was not sufficient to distinguish between carcinoma and mesotheliomas [3, 5]. Histopathological diagnosis to distinguish between metastatic carcinomas and mesotheliomas in human pleural lesions was applied by using a combination of E-cadherin and calretinin [6]. However, it also has the limitation of being invasive to humans or animals. In veterinary field, vimentin, E-cadherin, pancytokeratin, Wilms tumor 1 (WT1), MUC-1, and calretinin were used to differentiate between reactive mesothelial cells, malignant mesotheliomas, and adenocarcinomas in large felids only through post-mortem [7].

As clinicopathological diagnosis using five markers (cytokeratin, vimentin, desmin, E-cadherin, and calretinin) has not been previously reported in veterinary medicine, herein, the author presents a case of differential diagnosis using five markers in the pericardial fluid obtained with a minimally invasive method under ultrasound guidance [7–10].

## Case presentation

A 2.1 kg, spayed female, 10-year-old Yorkshire Terrier dog presented to a local hospital with dyspnoea. The presence of pericardial fluid was confirmed on ultrasound, which was then collected under ultrasound guidance, and the physical and chemical properties of the pericardial fluid were examined to determine its characteristics. The bacterial and fungal culture tests yielded negative results, indicating no growth. There were no abnormalities in complete blood count and serum chemistry.

The patient was referred to Kangwon National University Veterinary Teaching Hospital. Total 30 ml of pericardial fluid was collected from the area where it was observed under ultrasound guidance with local anaesthesia; 1 mg/kg (0.2 ml) of alfaxalone was intravenously administered, followed by an additional 0.1 ml. After 10–15 min, 4 ml of 2% lidocaine diluted in saline (1:1) was administered. Bloody pericardial effusion was smeared and cytological examination was performed.

**Fig. 1:**
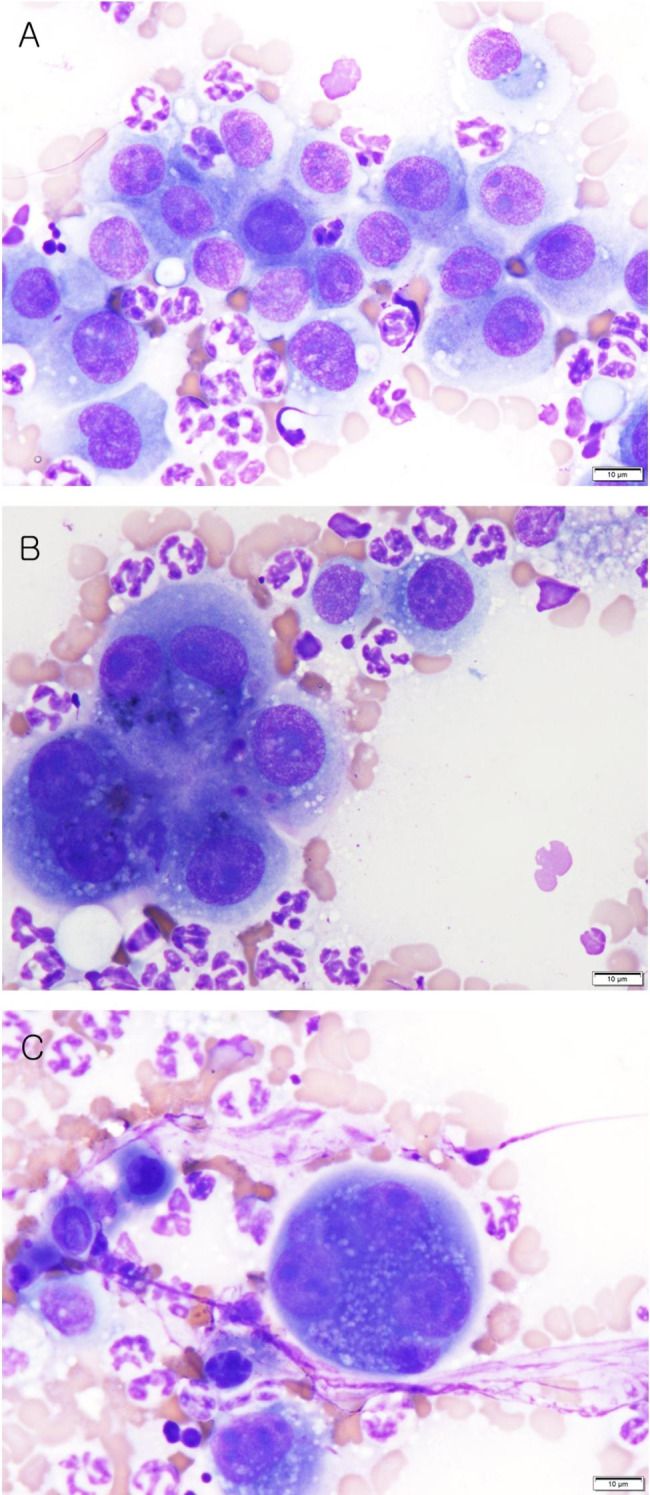
Cell smears from pleural effusion. (**A**) The cells showed anisocytosis, anisokaryosis, and abnormal nucleoli (large, angular, and multiple). (**B**) Atypical binucleated cells with distinct multiple nucleoli and numerous neutrophils were observed. (**C**) Large atypical multinucleate cells with multiple nucleoli and numerous neutrophils were observed (Diff-quick stain, ×1000, scale bar: 10 μm)

In cytological examination, binucleation, anisocytosis, anisokaryosis, abnormal nucleoli (large, angular, and multiple), abundant basophilic cytoplasm, high nuclear–cytoplasmic ratio (N:C), and coarse chromatin, and large atypical multinucleate cells, indicating high-grade malignancy were observed. These cells can be found either as individual or as clumps of aggregated with a number of erythrocytes. Numerous non-degenerative neutrophils and macrophages were observed alongside these cells. Erythrophagia was observed, indicating chronic hemorrhage (Fig. 1A–C).

Distinguishing between reactive mesothelial cells and mesothelioma in the pericardial fluid is challenging, especially in the presence of neutrophil-rich inflammation. Therefore, the author aimed to discover whether the observed cells were reactive mesothelial, mesothelioma, or adenocarcinoma cells via immunocytochemistry using five markers (cytokeratin, vimentin, desmin, E-cadherin, and calretinin) [3, 12–14].

Immunocytochemistry was performed by modifying the method recommended by the antibody manufacturers; mesothelioma cells as positive control for each of the primary antibodies were prepared from stored smear cells, which were collected from a dog patient diagnosed as mesothelioma post-mortem. At that time, the mesothelioma cells were smeared and fixed in methanol for 10 min and stored in a glass jar containing 1x phosphate-buffered saline (PBS) at 4 ℃ for several months.

For calretinin and E-cadherin staining, cell smears from pericardial effusions were fixed in methanol at -20 °C for 10 min and washed thrice with PBS (Sigma-Aldrich, Burlington, MA, USA) for 2 min. The primary antibodies against calretinin (1:1000, Sigma C7479; host: rabbit; reactivity: human, mouse, dog) and anti-E-cadherin, Alexa Fluor 594 (10 µg/ml, Biolegend 147,306; host: rat; verified reactivity: mouse, human; reported reactivity: cynomolgus, dog, pig), were diluted with 1% bovine serum albumin (BSA)/PBS (Komabiotech, Seoul, South Korea). Following overnight incubation with the primary antibodies at 4 °C, the slides were rinsed thrice in PBS for 2 min. The secondary antibody Alexa Fluor 488 (10 µg/ml, Invitrogen, A11034) goat anti-rabbit immunoglobulin G (IgG; heavy + light chain [H + L]) was diluted with 1% BSA/PBS. Following overnight incubation with the secondary antibodies at 4 °C, the slides were rinsed thrice in PBS for 2 min. The slides were then counterstained with mounting medium containing DAPI (Vector Laboratories, Burlingame, CA, USA) and examined using an LSM 780 laser-scanning confocal microscope (Carl Zeiss, Jena, Germany).

For cytokeratin and vimentin staining, pan cytokeratin monoclonal antibody (AE1/AE3), eFluor™ 570 (1 µg/ml, Invitrogen 41-9003-82, Carlsbad, CA, USA), vimentin monoclonal antibody (V9), and fluorescein (1 µg/ml, Invitrogen 11-9897-82) were used; the same protocol (staining method or time required) as described above was performed using a different secondary antibody.

For desmin staining, desmin monoclonal antibody (D33) (1:100, Invitrogen MA5-13259) was used as a primary antibody, and Alexa Fluor 488 (1:500, Invitrogen A28175) goat anti-mouse IgG (H + L) was used as a secondary antibody. The same procedures as those described above were performed.

**Fig. 2:**
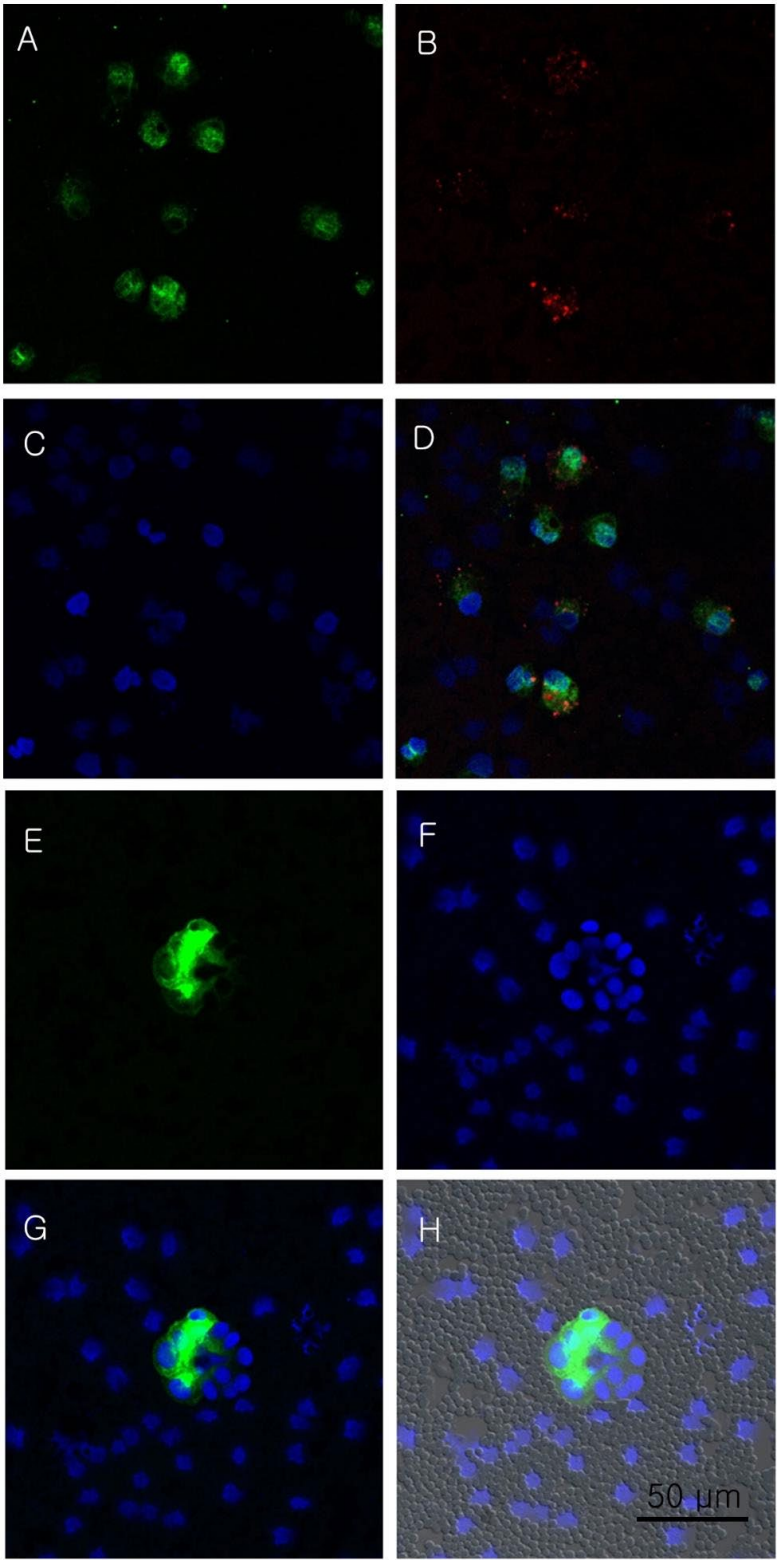
Immunocytochemistry for cytokeratin, vimentin, and desmin. (**A**) Vimentin (green), (**B**) cytokeratin (red), (**C**) DAPI (blue), (**D**) merged image of A, B, C, (**E**) desmin (green), (**F**) DAPI (blue), (**G**, **H**) merged image of E, F (×400, scale bar: 50 μm)

**Fig. 3:**
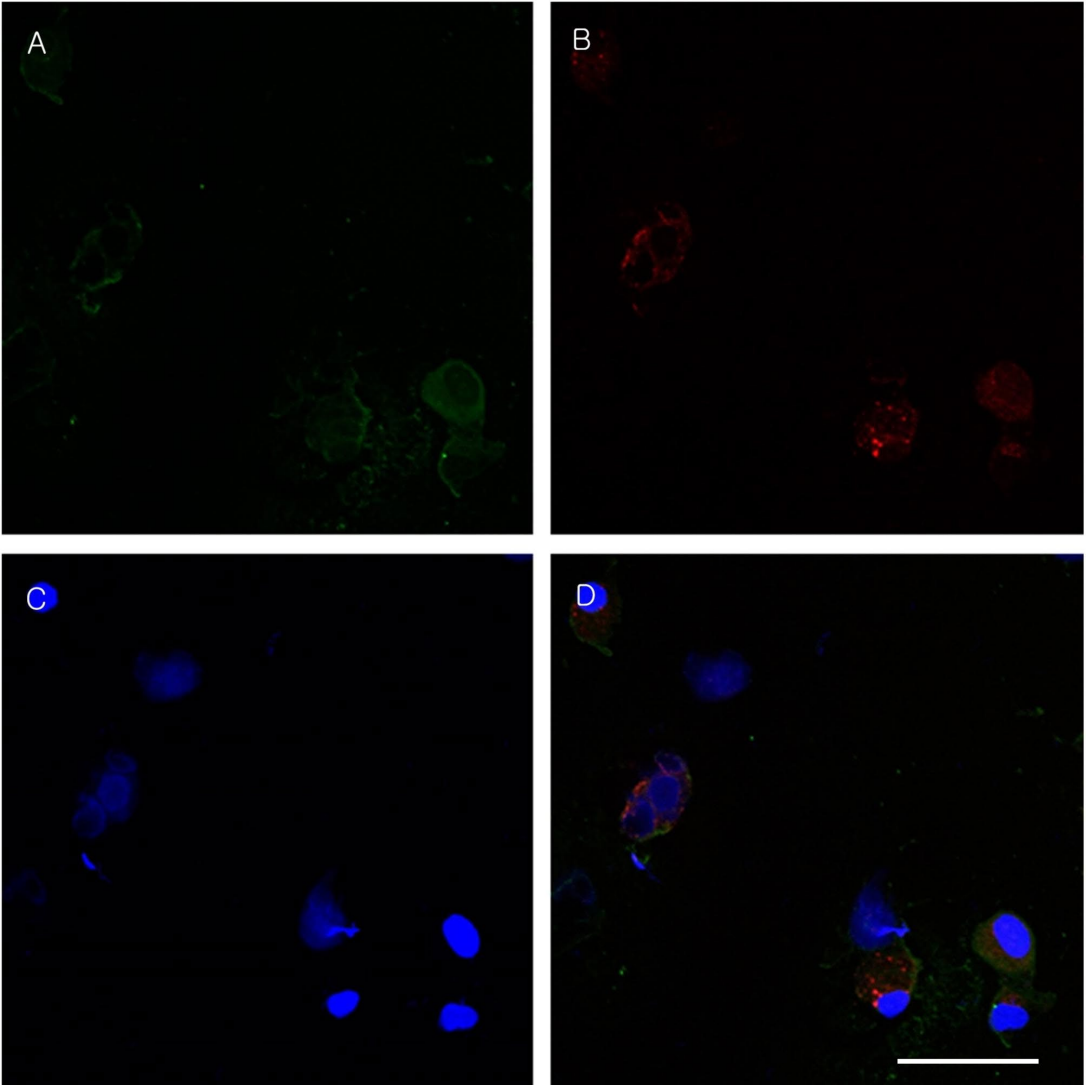
Immunocytochemistry for E-cadherin and calretinin. (**A**) Calretinin (Green), (**B**) E-cadherin (red), (**C**) DAPI (blue), (**D**) merged image of A, B, C (×400, scale bar: 50 μm)

Immunocytochemistry of pericardial fluid was positive for vimentin (Fig. 2A) and cytokeratin (Fig. 2B). DAPI (Fig. 2C) and the merged image of Fig. 2A–C are presented in Fig. 2D. It was also positive for desmin (Fig. 2E). DAPI (Fig. 2F) and the merged image of Fig. 2E, F are shown in Fig. 2G, H. Finally, it was positive for calretinin (Fig. 3A) and E-cadherin (Fig. 3B). DAPI (Fig. 3C) and the merged image of Fig. 3A–C are shown in Fig. 3D. Therefore, malignant mesothelioma was diagnosed [3, 10, 12–14].

## Discussion and conclusions

Common causes of haemorrhagic pericardial effusion include neoplasia, such as hemangiosarcoma, mesothelioma, chemodectoma, and ectopic thyroid tumours, and benign idiopathic pericardial effusion [1].

Cytokeratin is a well-known marker for epithelial cells, whereas vimentin is a marker for mesenchymal cells [11]. Furthermore, desmin is a muscle-specific protein and a key subunit of the intermediate filament in cardiac, skeletal, and smooth muscles. In a previous canine study, 100% of reactive mesothelial cells and malignant mesothelioma cases were positive for cytokeratin, vimentin, and desmin [11]. In contrast, adenocarcinoma cases were positive for cytokeratin, negative for desmin, and generally negative for vimentin, but some cases showed nonspecific vimentin positivity; sarcoma cases were positive only for vimentin [11]. As such, immunocytochemistry using cytokeratin, vimentin, and desmin could differentiate between malignant mesothelioma and adenocarcinoma, but not between reactive mesothelial cells and malignant mesothelioma.

Although E-cadherin and calretinin has not been used for pericardial effusion in dogs, it appears to be useful in distinguishing between reactive mesothelial cells and malignant mesothelioma from the literature search [6, 7]. E-cadherin is specifically expressed on epithelial cells and is a member of the Ca2+-dependent cell adhesion molecule family [12]. Calretinin is a 29 kDa calcium-binding protein expressed in neuronal cells. Recent studies using immunohistochemistry revealed that calretinin is a very useful marker for both reactive and neoplastic mesothelial cells [13, 14].

Immunostaining using calretinin showed positive results in 100% of reactive mesothelial cells and malignant mesothelioma cases, but it was not stained in adenocarcinoma cases. Conversely, in immunostaining using E-cadherin, 100% of malignant mesothelioma and 87% of adenocarcinoma cases were stained positive, but reactive mesothelial cells were not stained [3]. As such, in many human cases, it was confirmed that reactive mesothelial, malignant mesothelioma, and adenocarcinoma cells could be distinguished using two markers: E-cadherin and calretinin (reactive mesothelial cells: E-cadherin negative, calretinin positive; malignant mesothelioma: E-cadherin positive, calretinin positive; adenocarcinoma: E-cadherin positive, calretinin negative).

In this study, immunocytochemistry using pericardial fluid was cytokeratin-positive, vimentin-positive, desmin-positive, E-cadherin-positive, and calretinin-positive. Therefore, malignant mesothelioma was diagnosed [3, 10, 12–14].

Immunocytochemistry is a very useful diagnostic technique because it can determine whether several fluorescent markers are simultaneously expressed in the same cell. This study revealed that two well-established markers in humans and six large felids, E-cadherin and calretinin, can be used for the differential diagnosis of reactive mesothelial cells, malignant mesothelioma, and adenocarcinoma in dogs. In previous studies, an autopsy was performed to diagnose the animal post-mortem, and samples from a visible lesion were biopsied and immunostained (histopathological diagnosis). However, in this study, pericardial fluid was collected using a minimally invasive method under ultrasound guidance for the diagnosis of disease on an alive patient (clinicopathological diagnosis). Furthermore, diagnostic accuracy and time efficiency can be improved due to simultaneously checking multiple antigens within a same slide.

As far as the author knows, there haven’t been any commercial labs in this area that provide the immunocytochemistry diagnostic test the author conducted so far. The immunocytochemistry diagnostic test took two days to complete. The estimated cost of the immunocytochemistry diagnostic test would be hundreds of dollars per marker. With the help of the five markers, a definitive diagnosis could be possible in all cases. Although the test with fewer markers (E-cadherin and calretinin) is believed to provide definitive diagnosis in all cases, further studies with larger sample sizes are needed.

## Data Availability

All datasets are available in the main manuscript.

## Abbreviations

N: C:nucleus–cytoplasm ratio
KDa: kilodalton
PBS: phosphate-buffered saline
BSA: bovine serum albumin
H + L: heavy + light chain
FITC: fluorescein
